# From research to real-life implementation: an evaluation of the scale up of a guided digital mental health intervention in Lebanon: Step-by-Step

**DOI:** 10.3389/fpubh.2025.1665093

**Published:** 2025-11-11

**Authors:** Jinane Abi Ramia, Sally Khoury, Narsis Armani, Pim Cuijpers, Kenneth Carswell, Edith van ‘t Hof, Edwina Zoghbi, Marit Sijbrandij, Rabih El Chammay

**Affiliations:** 1 Department of Clinical, Neuro and Developmental Psychology, WHO Collaborating Center for Research and Dissemination of Psychological Interventions, Amsterdam Public Health Research Institute, Vrije Universiteit Amsterdam, Amsterdam, Netherlands; 2 National Mental Health Programme, Ministry of Public Health of Lebanon, Beirut, Lebanon; 3 International Institute for Psychotherapy, Babeş-Bolyai University, Cluj-Napoca, Romania; 4 Department of Noncommunicable Diseases and Mental Health, World Health Organization, Geneva, Switzerland; 5 Country Office for Lebanon, World Health Organization, Beirut, Lebanon; 6 Psychiatry Department, Faculty of Medicine, Saint Joseph University, Beirut, Lebanon

**Keywords:** digital mental health, implementation science, RE-AIM, Step-by-Step, Lebanon, LMIC, scale-up, uptake

## Abstract

**Introduction:**

Digital mental health interventions offer a promising approach for addressing the global mental health treatment gap. However, concerns remain regarding their effectiveness and scalability in real-world settings, particularly in crisis-affected low-and middle-income countries (LMICs). Following randomized clinical trial research, the World Health Organization’s Step-by-Step (SbS) guided digital self-help intervention for depression and anxiety was scaled up in a pilot implementation by the National Mental Health Programme (NMHP) in Lebanon. This paper outlines results from this pilot and its integration into healthcare in a setting facing political, financial, and humanitarian crises.

**Methods:**

A mixed-methods implementation study using the RE-AIM framework assessed the reach, effectiveness, adoption, implementation, and maintenance of SbS in Lebanon. Quantitative analyses evaluated clinical, satisfaction, and uptake outcomes. Forty two key informant interviews with SbS users, staff, and key stakeholders assessed the public health impact and implementation success in Lebanon.

**Results:**

Despite contextual challenges affecting SbS’s reach and adoption, findings indicate that SbS had high uptake with 1,942 users completing the baseline assessment. Significant reduction in symptoms of depression was observed with an effect size of *r* = 0.69 (pre and post-tests). With a high dropout rate (62% of the starters), qualitative findings explored strategies to optimize user adherence, such as improving in-app engagement and early rapport-building. Acceptability and adoption among disseminating partners were evident yet concerns prevailed around the team’s capacity and the ability to manage risk remotely. By the end of the pilot, SbS had not yet been formally integrated into the mental health referral model but was instead provided as a national service signposted by several facilities. While global interest and funding for digital interventions present an opportunity for sustaining SbS, barriers include poor internet access, lack of sustainable financing, and the absence of a comprehensive referral model.

**Discussion:**

Results suggest that SbS has the potential to provide evidence-based treatment for depression across the whole of Lebanon, potentially as a first-step treatment within the model in primary care. Reach can be expanded through social media, mass media, and outreach. Long-term sustainability will depend on securing stable funding, robust and formal integration models, and enhancing user adherence.

**Clinical trial registration:**

The initial clinical trial registration of the RCT phase was ClinicalTrials.gov, identifier NCT03720769.

## Introduction

Digital mental health interventions, including self-help, are emerging as an effective, cost-efficient, and widely scalable tool for treating common mental health disorders, notably depression and anxiety. Evidence is compelling about the potential of these interventions in addressing the mental health treatment gap worldwide (1–3). Recent studies from low- and middle- income countries (LMICs) echoed similar results among refugees and people affected by adversities (4, 5). Nevertheless, the question remains whether these interventions continue to be effective, engaging, and scalable in real-life practice beyond research trials. Studies show that the attrition rates in digital mental health interventions increase substantially during implementation phases. A systematic review found that the engagement rates in 10 unguided digital-mental health interventions were 4.06 times higher in trial phases than in real-world settings for the same programs (6). Another review showed that indications of sustained engagement in unguided digital mental health interventions during or after 6 weeks of completion in real-world settings range from 0.5 to 28.6% as compared to an adherence rate of 50–100% in research studies (7). As for guided interventions, studies have consistently reported a higher engagement rate than unguided interventions- up to 47% completion rate compared to 29% in unguided- and identified human support as a key promoting factor for adherence (8, 9). Nevertheless, guided interventions too, often witness higher engagement rates in research trials than in real-world scenarios (10). Potential predictors of higher engagement during controlled trials include the provision of financial compensation, strict inclusion/exclusion criteria, the rigid follow-up procedures, and the highly supervised care delivery (6, 9). In contrast, real-life implementation presents multilevel challenges such as stigma, inaccessibility to smartphones, SIM cards or internet, low literacy, confidentiality and data security concerns, and organizational obstacles such as inadequate referral and integration models within the healthcare setting (11, 12). For these reasons, among others, scaled-up digital mental health interventions often witness low uptake, and there is limited evidence around their scalability models (13). It is thus essential to conduct more implementation studies on the scalability of digital mental health interventions in real-life settings to assess their efficacy and identify challenges and solutions for uptake. These evaluations would help shape better scale-up models, strengthen the integration of digital mental health interventions in general healthcare, and increase their access among vulnerable groups, refugees, and those affected by adversities (8).

Step-by-Step (SbS) is a digital mental health intervention for depression in adults (14). It is one of many scalable psychological interventions, designed for delivery by non-specialists, developed by the World Health Organization (WHO) to increase accessibility to mental healthcare worldwide, including LMIC. SbS is a five-week digital self-help program available on a phone application or website, with minimal guidance provided by trained and supervised non-specialists called e-helpers. These e-helpers offer a 15-min weekly support contact via phone or messaging (14). The project was developed in collaboration with the National Mental Health Programme (NMHP) at the Ministry of Public Health (MoPH) and other partners in Lebanon (15). The first version of the SbS intervention was adapted to local communities in Lebanon through a cultural adaptation (16). SbS underwent extensive testing in Lebanon through an uncontrolled pilot (17), a feasibility randomized controlled trial (RCT) (18), and two definitive RCTs for Syrian refugees and the Lebanese host community in Lebanon (4, 5). The acceptability, feasibility, and uptake in the research trials were assessed through a qualitative evaluation of the RCTs (19) and cost-effectiveness was proven in a subsequent study (20). In parallel, the SbS intervention was culturally adapted and tested for Syrian refugees in Egypt, Germany, and Sweden as part of the STRENGTHS project (12). SbS adaptation and effectiveness studies have also been conducted among Albanian refugees in Switzerland, university students in China, and overseas Filipino workers in Macao (21–24). After proving its effectiveness and cost-effectiveness (4, 5, 20), SbS was scaled-up in Lebanon through a phased approach, with an 18-month (April 2021–October 2022) pilot-scale up before the full-fledged scale-up. The aim of the pilot-scale up was to test the feasibility and effectiveness of SbS in a real-life setting in Lebanon outside of the RCTs, and to assess the success of the implementation model and integration into the existing health care system. The RE-AIM framework was used to evaluate this implementation pilot. RE-AIM is typically used to evaluate the implementation of innovative interventions into existing healthcare settings by assessing the reach, effectiveness, implementation success, adoption level, and maintenance of the intervention (22, 25). RE-AIM was also used to evaluate the feasibility and implementation of SbS in its early research phases in China and Macao (21, 24).

Lebanon is a lower-middle income country in the Middle East that has experienced multifaceted crises at the political, economic, and public health levels. It is known for hosting one of the highest ratios of Syrian refugees per capita globally (26). Mental disorders are on the rise in Lebanon, with one in four persons suffering from a mental disorder, whereas only 10% have access to adequate care (27, 28). The situation has worsened with multiple overlapping crises, including the Beirut port explosion, COVID-19 pandemic, the economic collapse, political instability, and the recent war on Lebanon. Mental disorders have surged, with nearly half the population in 2022 screening positive for depression (47.8%), anxiety (45.3%), and post-traumatic stress disorder (PTSD) (43.5%) (29, 30). Despite the progress made by NMHP and partners in reforming the mental health system in Lebanon (31), access to evidence-based and affordable mental health services remains a significant challenge (32). Innovative solutions such as SbS may help bridge the mental health treatment gap. This pilot implementation study was conducted to test the scalability of SbS in Lebanon. The study attempts to answer the following questions (1) To what extent do guided self-hep digital mental health interventions like SbS maintain their effectiveness, uptake, and public health benefits beyond research trials among people affected by adversities? (2) To what extent was the adoption and implementation of SbS successful among facilities in Lebanon and what are the opportunities and challenges encountered in real-life setting? and (3) What are the barriers to the integration and long-term sustainability of digital mental health interventions in Lebanon’s healthcare system and what strategies could mitigate them? This study was conducted to guide the full scale-up of SbS and inform a model for sustainable delivery of SbS in Lebanon and potentially other countries.

## Methods

### Participants and procedures

**Figure 1:**
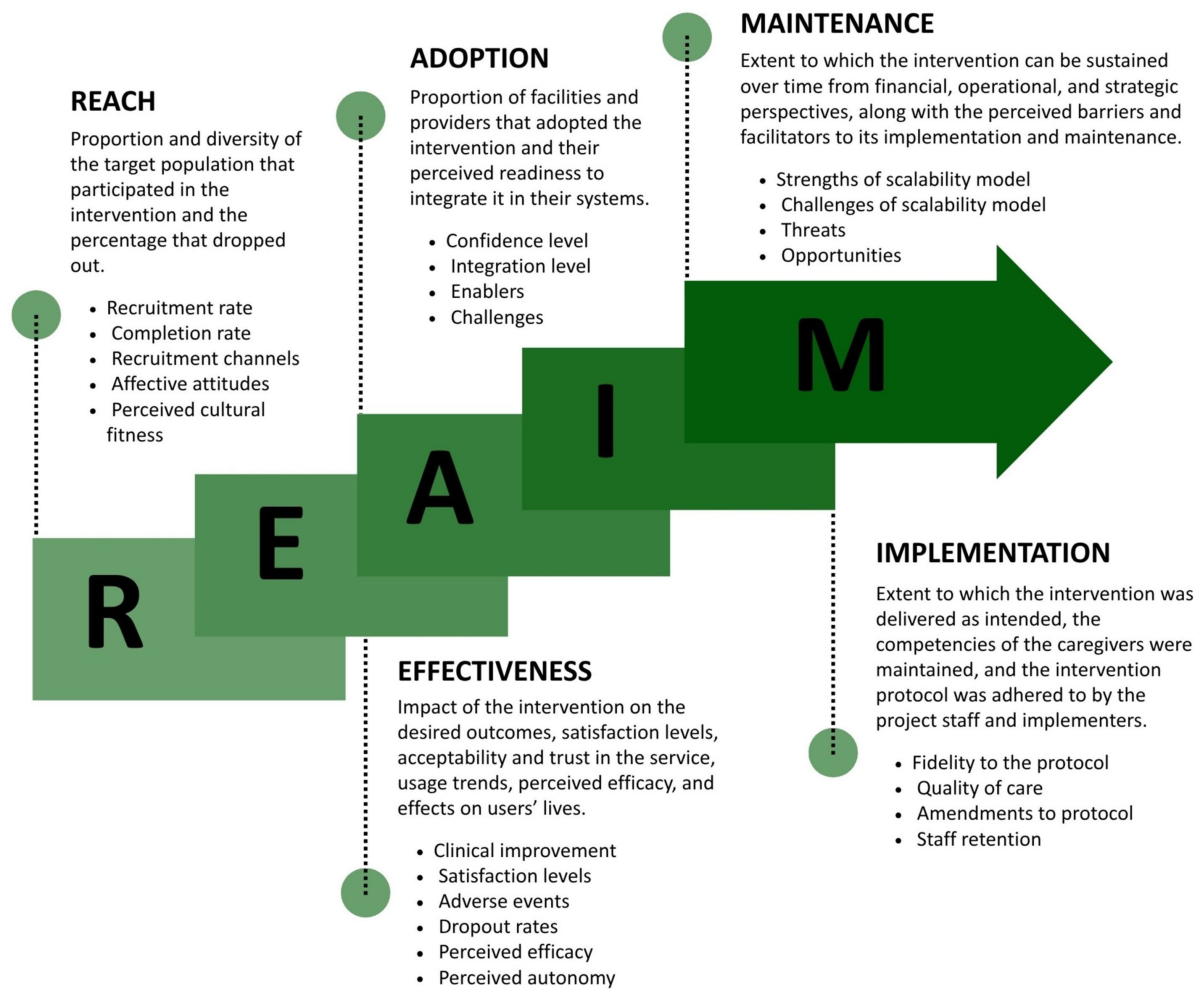
RE-AIM framework and indicators.

The study is a quasi-experimental implementation study that uses a mixed-method approach to evaluate the pilot-scale up of SbS in a non-research, real-life setting in Lebanon. Participants were eligible to access the intervention if they were 18 years or older, living in Lebanon, and scored 10 or above on the PHQ-9 at baseline, indicating at least moderate depressive symptoms. The RE-AIM (Reach, Effectiveness, Adoption, Implementation, and Maintenance) evaluation framework (32), was used to capture the effectiveness, uptake, and implementation model. This framework was chosen given its relevance and wide acceptance for testing pragmatic models and web-based technologies (33, 34). The framework illustrated in Figure 1 assumes five domains to assess the success and generalizability of a new intervention into an existing system (1) Reach, (2) Effectiveness, (3) Adoption, (4) Implementation, and (5) Maintenance (33).

A mixed-method evaluation approach was used to assess the different domains (25, 35). In the RE-AIM framework, *Reach* was assessed by evaluating the number of users who signed up, their demographic distribution, the completion rates, and the efficacy of different recruitment channels. Qualitative interviews depicted the acceptability factors of SbS, the affective attitude toward the intervention, and its perceived match to the local culture. *Effectiveness* was assessed by evaluating improvements in users’ clinical symptoms, satisfaction levels, adverse events encountered, and drop-out rates. Users’ perceived efficacy of SbS was assessed by exploring their confidence in performing the tasks and adopting the behavioral changes required to manage their depression. Findings were triangulated from qualitative interviews, quantitative analyses of responses to the Patient Health Questionnaire, PHQ-9 (36, 37), World Health Organization Disability Assessment Scale 2.0, WHODAS (38) and Generalized Anxiety Disorder-7, GAD-7 (39), as well as user satisfaction questionnaires (40). *Adoption* was examined by assessing the level of confidence among partners to adopt and disseminate SbS and by looking into potential obstacles for future adoption. *Implementation* was evaluated based on the extent to which SbS was delivered as originally planned. This was done by assessing the average length of time to complete the program, e-helpers’ adherence to the protocol, duration of the support sessions, staff retention rates, and competency assessments of e-helpers. *Maintenance* was explored by gathering stakeholders’ feedback on the different challenges, opportunities, and contextual threats to the sustainability of SbS in Lebanon. Recommendations were collected for each of the five domains.

A qualitative process evaluation took place with a total of 42 interviews; those were divided into 20 SbS users and all seven e-helpers, a clinical supervisor, a senior e-helper, a project coordinator (with the last three referred to as supervisors to ensure confidentiality), and 12 external stakeholders (policy makers, project partners, and disseminating partners). E-helpers asked all users for consent to participate in the interviews upon signing up to the program. To ensure a diverse range of participants, an independent researcher used a stratified random sampling method in Statistical Package for the Social Sciences (SPSS) v26 Software, to generate a random sub-sample of users stratified by nationality (Lebanese/Syrian), gender (Male/Female), completion status (completed/dropout), and preferred support method (message/call). E-helpers reached out to users by phone for another round of oral consent. Table 1 reports demographic data about the users who were interviewed. Table 2 details the distribution of the project staff and external stakeholders interviewed. A purposeful sampling technique was used to interview external stakeholders, depending on their involvement in the project and their availability. Stakeholders included partnering non-governmental organizations (NGOs), United Nations (UN) agencies, project counterparts from NMHP and WHO, and universities that integrated SbS into their set of referral services. Organizations and job titles were not mentioned in the results section to maintain confidentiality.

**Table 1:** Demographic data of key informant study participants stratified by completion status.

Demographic variables	SbS participants interviewed (*n* = 20)	
	Completed SbS (*n* = 17)	Dropped out SbS (*n* = 3)
Age, *M* (SD)	Mean 29.8 (8.11)	Mean 28.33 (9.74)
Sex, *n* (%)	Male = 7(41%) Female = 10 (59%)	Male = 0 (0%) Female = 3 (100%)
Nationality, *n* (%)	Lebanese = 14 (82.35%) Syrian = 3 (17.65%)	Lebanese = 2 (66.67%) Syrian = 1(33.33%)

*Dropout: study dropouts who either proactively asked to be dropped out or discontinued and did not complete the post-assessments.

**Table 2:** Overview of key informants stratified by different stakeholder groups, positions, and sex.

Demographic variables	SbS project staff and external stakeholders interviewed (*n* = 22)	
	SbS project staff (*n* = 10)	External stakeholders (*n* = 12)
Sex, *n* (%)	Male = 4 (40%) Female = 6 (60%)	Male = 2 (17%) Female = 10 (83%)
Organization	National Mental Health Programme	American University of Beirut (AUB) Embrace International Medical Corps (IMC) Lebanese American University (LAU) National Mental Health Programme (NMHP) United Nations High Commissioner for Refugees (UNHCR) World Health Organization Lebanon (WHO-Leb) World Health Organization Head Quarters (WHO-HQ)
Position	Project Coordinator Clinical Supervisor Senior E-helper E-helper	Community-Based Protection Officer Director of Business Support Director of Counseling Center Director of Strategic Partnerships Founder and Director Head of Program Media Advisor Mental Health Coordinator Mental Health Technical Officer National Officer for Noncommunicable Diseases and Mental Health Operations Manager Principal Counselor

The project coordinator and senior e-helper conducted in-depth interviews with the users and stakeholders, while research assistants from MoPH, independent from the project, conducted interviews with project staff and some of the stakeholders. All interviewers were trained by the project coordinator on the semi-structured interview guides prior to the evaluation. An email was sent by the NMHP to all stakeholders and staff inviting them to participate in the interviews and written consent was obtained via email. For users, as there was no in-person contact, and due to limited technological literacy or absence of email addresses for some, information on the purpose of the interview and oral consent to be contacted was sought by their corresponding e-helper at the end of the final support session. On the day of the interview, the researcher read the interview information and consent form aloud and obtained recorded oral consent before starting the interview (Supplementary material S1). Phone or online interviews on Teams or Zoom were conducted and lasted 45 min on average and were recorded and translated into English by the project coordinator, senior e-helper, and the MoPH research assistants.

Semi-structured interview guides were developed and tailored for each group of interviewees to capture the different indicators. For stakeholders, nine questions were designed to gather their feedback on reach, adoption, and maintenance; seven additional questions were asked to those who hosted the project as partners (Embrace) (Supplementary material S2). For staff, the guides comprised 23 questions that inquired about the implementation of SbS in the naturalistic world (Supplementary material S3). For users, the guides were 24 questions-long and tackled the reach and effectiveness domains (Supplementary material S4).

### Analysis

**Figure 2:**
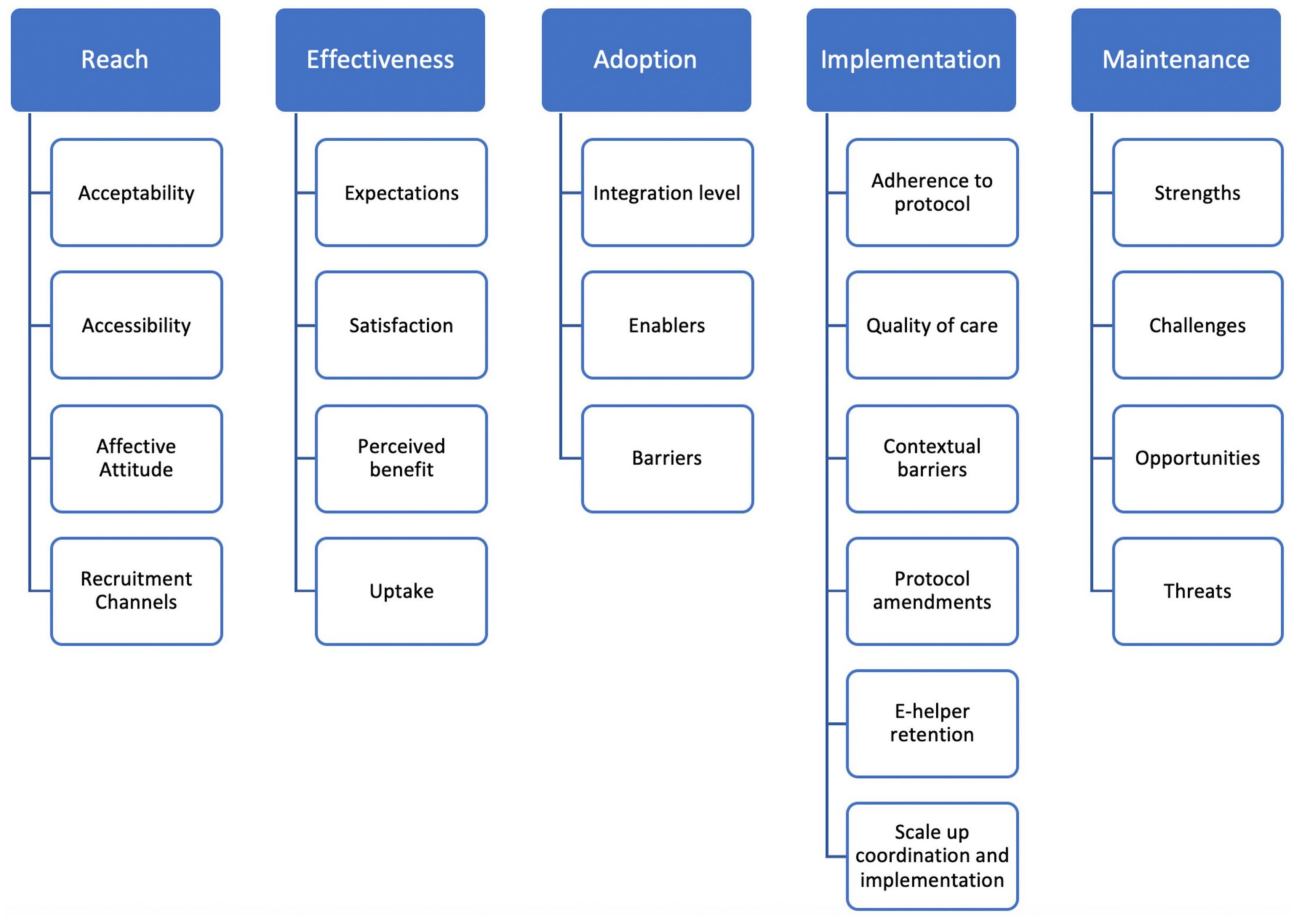
Thematic tree of the Step-by-Step qualitative evaluation analysis.

Given the non-normal distribution of data, Wilcoxon signed-rank tests were used to assess the pre-post tests of depression (PHQ-9), anxiety (GAD-7), functioning (WHODAS 2.0), and the satisfaction level (Client Satisfaction Questionnaire, CSQ-3). To measure effect size, r = Z/√N was used where Z is the standardized test statistic value from the Wilcoxon test and N is the total number of observations. Equivalent Cohen’s D effect sizes were computed to enable comparison across studies, by dividing the difference between two means by the pooled standard deviation. Effect sizes were considered as small (*d* = 0.2), medium (*d* = 0.5), and large (*d* = 0.8) (41). The response to treatment and remission rates were calculated to measure improvements in clinical symptoms. Response to treatment is defined as a >50% reduction in baseline PHQ-9 scores. Remission is defined as scoring <5 on the PHQ-9 post-test, meaning users no longer exhibited symptoms of depression. Descriptive analyses reported on demographic and usage metrics. Additional tests such as Wilcoxon Signed Ranks Test and Kruskal–Wallis H test were performed to assess significance in improvement within groups of different depression severity levels at baseline and to examine whether improvement in scores differed by baseline depression severity (41). The project coordinator (first author) conducted a thematic analysis of the transcripts via the Nvivo 2017 software for qualitative analysis using the deductive framework approach, which consisted of following pre-determined themes from the interview guides. The themes inquired about in the interview guides are presented in Figure 2 and are congruent with the different domains and indicators of the RE-AIM framework. After familiarization with the transcripts, the coordinator generated codes under each of these themes. The coordinator analyzed each key-informant sub-group separately, and triangulated data from all groups to report commonalities and divergences in responses and categorized them under each theme and domain of the RE-AIM framework (42). The senior e-helper (second author) performed a random cross-checking of sample transcripts to validate the codes. As a start, key-informant groups were analyzed separately, then common findings emerging under the same domains of the RE-AIM framework were compared and reported jointly under each domain.

### The intervention

Step-by-Step is a digital mental health intervention for the treatment of depression and anxiety among adults. It is a self-help brief 5-week program available on a website or a smartphone application. It uses several therapeutic techniques for the treatment of depression and anxiety symptoms, such as behavioral activation, stress management, social support, and relapse prevention. These techniques are delivered through an illustrated story of a character, along with interactive activities in between the weekly sessions. Activities include small self-care activities, complex activities that can be broken down into smaller steps, relaxation techniques such as slow breathing and grounding exercises, gratitude list, positive self-talk, affirmations, regaining social interactions and identifying warning signs. There is also a mood tracker and an online calendar to schedule activities. In Lebanon, SbS was delivered in Arabic and English with minimal remote guidance by trained and supervised non-specialists called e-helpers, who deliver phone or message support for 15 min per week (14). E-helpers follow a structured outline and a manual for support and are supervised by a clinical psychologist through individual and group meetings on a weekly basis. Clinical improvement is measured by self-administered baseline and post assessments embedded in the application. SbS was culturally adapted and tested in Lebanon over a period of 5 years. After proving effective and cost-effective, it was scaled up into a national service, that is free to users. The pilot scale-up project aimed at testing the delivery model and the integration of SbS into the existing health care system over a period of 18 months (April 2021–October 2022), under the management of NMHP, and the hosting of a local NGO called Embrace, who also operates the National Lifeline for Emotional Support and Suicide Prevention in Lebanon in collaboration with NMHP (43). Improvements to the application and protocol were made, and 12 e-helpers were recruited, trained, and supervised to deliver the intervention, six of whom were retained throughout the pilot and one more subsequently joined. Participants were recruited through social media messages on the NMHP official Facebook and Instagram pages. Other NGOs in the Mental Health and Psychosocial (MHPSS) Support Coordination Group in Lebanon- a coordination group encompassing more than 130 actors providing MHPSS in Lebanon- universities, private organizations, and healthcare providers were approached to disseminate SbS among their beneficiaries. SbS was also integrated in all the national guidelines and official communication channels of NMHP.

## Results

### Reach

**Figure 3:**
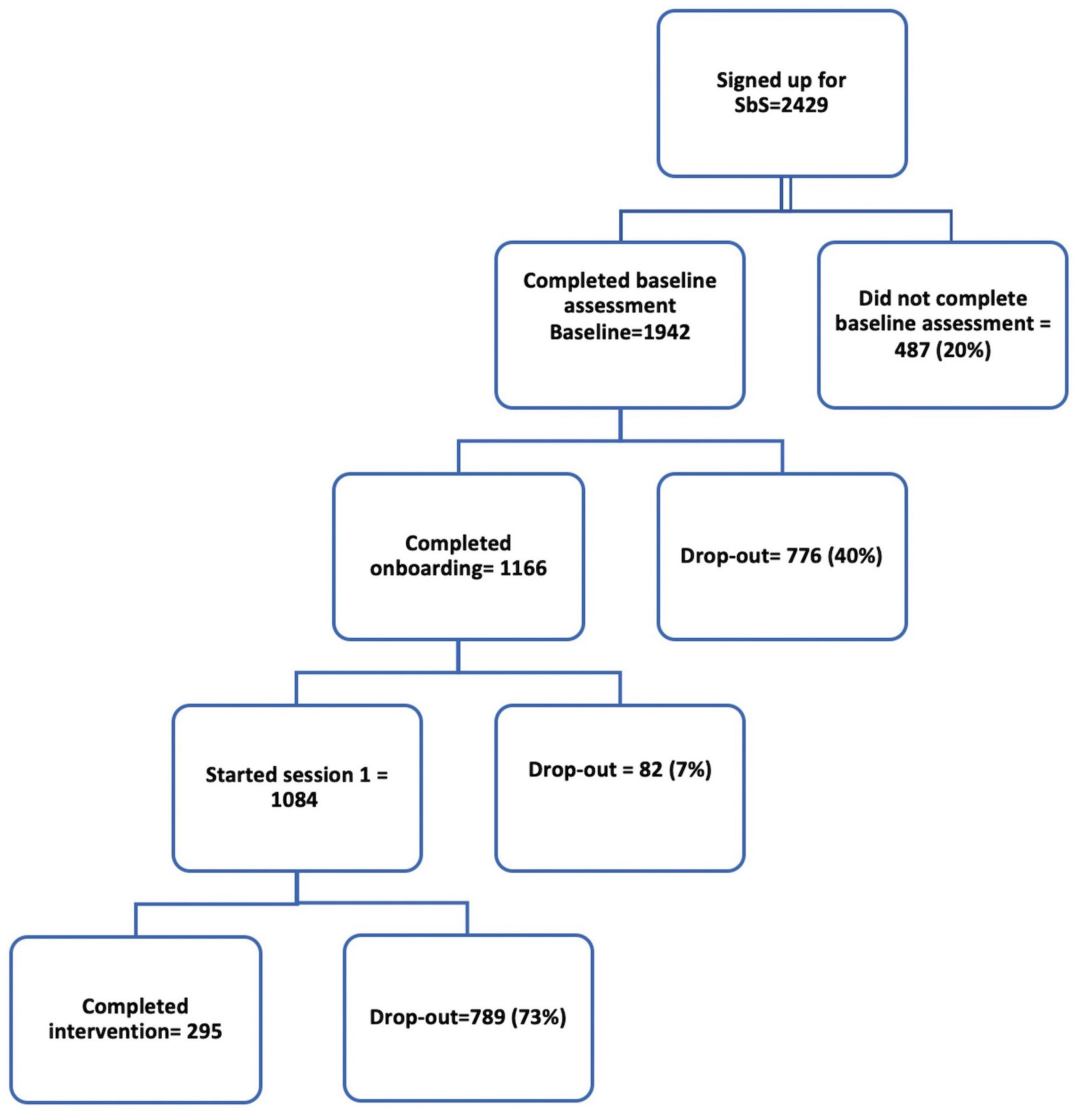
Flowchart of user enrolment and completion rates in SbS.

During the implementation study (April 2021–October 2022), 2,429 users were recruited, slightly exceeding the target of 2,400. A total of 1,942 people completed the sign-up process, of whom 1,166 completed the program introduction and were either assigned to an e-helper or were provided with no support, according to their preference. Program completion was defined as completion of four out of five sessions. Of those who completed the baseline assessment (1,942), 15.2% (295) completed the program. Among the “starters”- those defined as users who started session 1 part 2 (776) (e.g., people who have engaged long enough to get to this point in the program)-38% (295) completed the program. A higher number of people (355) completed pre and post-tests, even though some of these had not completed the program. Figure 3 shows the flow of recruitment and completion.

**Figure 4:**
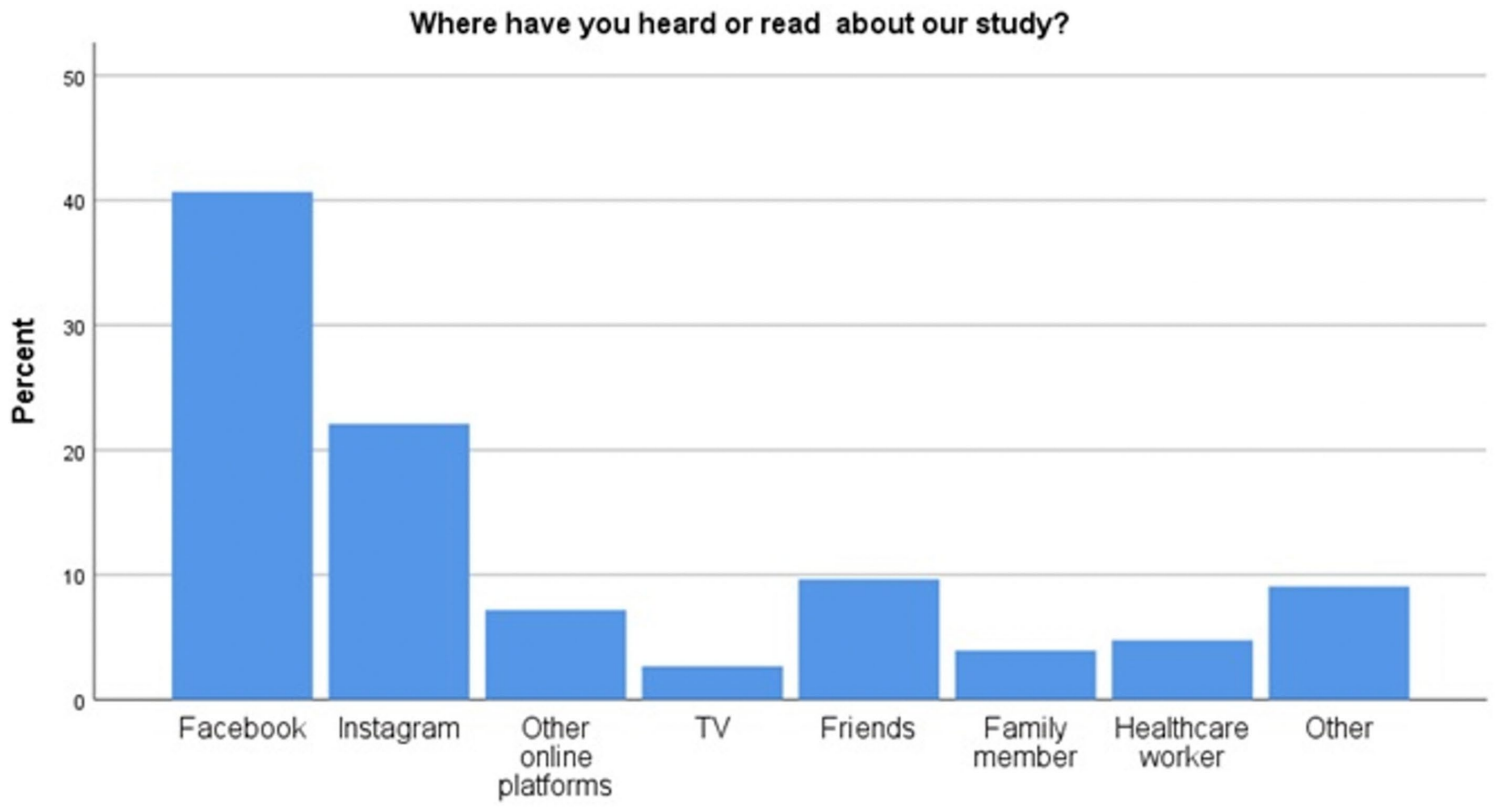
Bar chart showing the percentage of users recruited per channel or medium.

As shown in Figure 4, most of the users were recruited through social media, notably Facebook. Among these, the majority were young unmarried adults with a university degree and a paid job. Results on users’ demographics in Table 3 showed that more Lebanese than Syrians signed up to SbS through social media with only 11% of participants being Syrians.

**Table 3:** Demographics of users who participated in Step-by-Step.

Demographic information	Demographic variables	*n* (%)
Gender *N* = 1,870	Female	1,350 (72.2)
Age, *M* (SD)	Age Mean and SD	29.1 (9.2)
Age range *N* = 1,515	18–24	598 (39.5)
	25–38	697 (46.1)
	39–45	130 (8.6)
	46 and above	90 (5.9)
Preferred *N* = 1,870	Arabic	1,393 (74.5)
	English	477 (25.5)
Nationality *N* = 1,515	Lebanese	1,271 (83.9)
	Syrian	172 (11.4)
	Palestinian	49 (3.2)
	Other	10 (0.7)
	Would rather not say	13 (0.9)
Work status *N* = 1,513	Paid work (employee/self-employed)	622 (41.1)
	Non-paid work (volunteer, charity, homemaker)	108 (7.1)
	Student	312 (20.6)
	Retired	11 (0.7)
	Unemployed for other reasons	460 (30.4)
Marital status *N* = 1,513	Never married	932 (61.6)
	Currently married	457 (30.2)
	Divorced or separated	107 (7.1)
	Widowed	17 (1.1)
Highest level of education *N* = 1,505	No education	6 (0.4)
	Primary school (age 3–6)	21 (1.4)
	Elementary education (age 6–15)	103 (6.8)
	Secondary education (age 15–18)	238 (15.8)
	Undergraduate or BSc degree (age 18+)	680 (45.2)
	Graduate or MSc degree	326 (21.7)
	Higher university degree (Ph. D.)	22 (1.5)
	Technical secondary education (age 18+)	109 (7.2)
Preferred character description *N* = 1,336	Married	479 (35.9)
	Not married	857 (64.1)
Preferred contact method by E-helper *N* = 830	Call	472 (56.9)
	Message	295 (35.5)
	No contact	63 (7.6)
How did you hear about SBS? *N* = 1,490	Facebook	595 (39.9)
	Instagram	355 (23.8)
	Other online platforms	113 (7.6)
	TV	48 (3.2)
	Radio	0 (0.0)
	Friends	133 (8.9)
	Family member	52 (3.5)
	Healthcare worker	55 (3.7)
	Other	139 (9.3)

All brackets reflect % except when specified otherwise.

Qualitative interviews revealed that the users’ acceptance of SbS was attributed to its convenience, ease of accessibility, toll-free aspect, confidentiality, perceived benefit, and their social network’s reactions to it. “*It was easier actually because it was on my own time. I love the idea that I can request which day is convenient [to receive e-helper support session]*” (Lebanese Female, intervention completer). Stakeholders observed that the younger, tech savvy adults from medium to high socio-economic status were more likely to access SbS while Syrians encountered more barriers such as low-literacy levels, limited access to the internet, severe problems and symptoms, and concerns about confidentiality breaches when using shared devices. Users expressed a strong emotional connection to the program and described it as safety and relief during crisis “*I felt like I was rescued by this application. It made me feel as if I am protected in a safe zone*” (Male, Lebanese, intervention completer). Users unanimously considered SbS to be a good cultural fit as it alleviated concerns of stigma, transportation, and financial burdens. Stories, illustrations, characters, and exercises resonated with their daily experiences and cultural definitions of mental health. Most users reported social media as the main recruitment medium (Facebook and Instagram). The word of mouth, the professional referral by mental health specialists in NGOs or private practice, and the proactive Google searches for mental health support were also mentioned as recruitment channels. Interviewees proposed a set of recommendations to enhance the reach of the SbS in Lebanon. These consisted of leveraging social and mass media and community outreach, developing engaging content, and fostering trust through personal stories and professional referrals.

### Effectiveness

SbS was shown to be effective for treating depressive and anxiety symptoms, measured 8 weeks after signing up. Significant treatment effects were shown for symptoms of depression, anxiety, and functioning where the mean score on the PHQ-9 decreased from 18.4 pre-intervention to 11.5 post-intervention (*Z* = −13.0, *p* < 0.001), with a large effect size (*r* = 0.69). As for the mean functioning score on the WHODAS, it decreased from 34.5 pre-intervention to 28 post-intervention (*Z* = −10.2, *p* = 0.001) with a moderate effect size (*r* = 0.55). The mean anxiety score on the GAD-7 decreased from 18 pre-intervention to 11.9 post-intervention (*Z* = −13.1, *p* < 0.001), with a large effect size (*r* = 0.72). Results are shown in Table 4. Equivalent Cohen’s D effect sizes, computed to allow estimates between studies were: PHQ9, d = 0.91 large effect, WHODAS, d = 0.63 moderate effect, and GAD 7, d = 0.96 large effect.

**Table 4:** Inferential statistics of treatment outcome measures for complete cases.

Assessment tools		Pre-assessment			Post-assessment					
	*N*	Median	Mean	SD (standard error mean)	Median	Mean	SD (standard error mean)	*Z*	*p* value	Effect size *r*
PHQ-9	355	18	18.4	4.84 (0.26)	10	11.5	7.3 (0.39)	−13.0	<0.001	0.69
WHODAS	342	34	34.5	9.38 (0.51)	26.5	28	10.03(0.54)	−10.2	<0.001	0.55
GAD-7	335	19	18	4.53 (0.24)	11	11.9	6.76 (0.37)	−13.1	<0.001	0.72

The response to treatment and remission rates were calculated to measure improvements in clinical symptoms. Response to treatment is defined as a >50% reduction in baseline PHQ-9 scores. Remission is defined as scoring <5 on the PHQ-9 post-test, meaning users no longer exhibited symptoms of depression. Of users who completed the post-test at the end of the program (*N* = 355), 41.1% showed treatment response and 46.8% were sub-threshold (PHQ-9 < 10) and 18.6% showed complete remission (PHQ-9 < 5) remitted.

**Figure 5:**
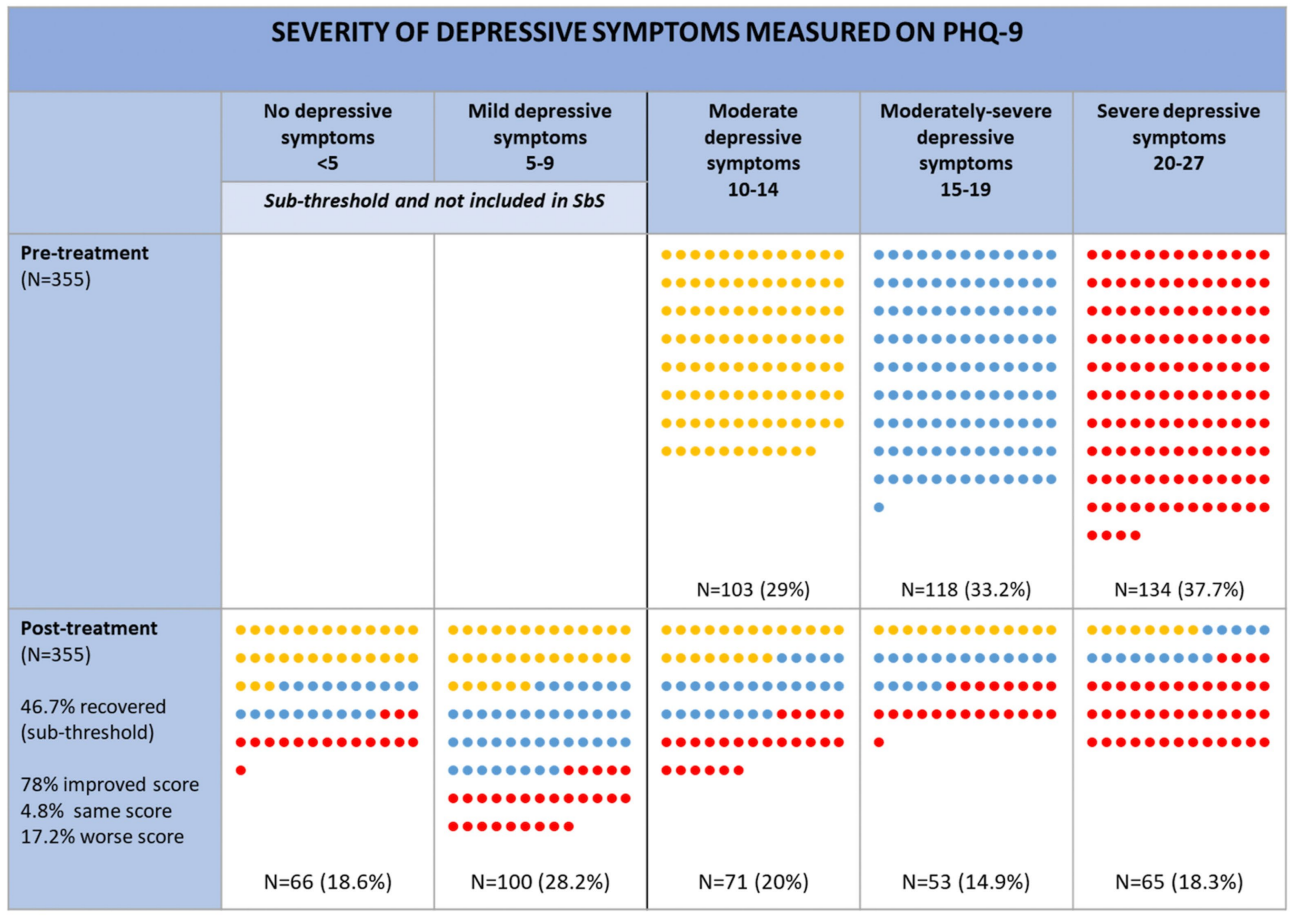
PHQ-9 categories of users pre- and post-SbS. Changes in score (e.g., improved score, same score, worse score, relates to the actual PHQ-9 score and not the category. E.g. someone may have an improved score but remain in the same category).

Figure 5 shows the severity of the depressive symptoms of users before and after they completed SbS. Around 38% of people showed severe depressive symptoms when they signed up for SbS, followed by 33% who reported moderate to severe symptoms and 29% who reported moderate symptoms. After the intervention, the percentage of people with severe, moderate to severe, and moderate symptoms were around 18, 15, and 20%, respectively. 17% reported increased symptoms and 5% reported the same amount of symptoms. In order to measure the movement between severity groups, Wilcoxon Signed Ranks Test was used and showed that the decrease in severity across groups was significant *p* < 0.001, *z* = −12.44, with a large effect size of *r* = 0.75. A Kruskal–Wallis H test was also conducted to examine whether improvement in PHQ-9 scores differed by baseline depression severity. The results indicated a significant difference among groups, H (2) = 28.45, *p* < 0.001.

Inspection of mean ranks showed that participants with severe baseline depression (mean rank = 207.08) experienced greater improvement than those with moderately severe (mean rank = 181.85) or moderate (mean rank = 135.75) depression at baseline.

A total of 66 users (5.6% of 1,166 who completed onboarding) were classified through e-helpers’ assessments as being at risk of Sexual and Gender-Based Violence, child protection case, or suicide. Of this number, six reported a serious adverse event (e.g., imminent risk of suicide) and were referred to specialized support. All at-risk users were encouraged to continue SbS and most did and were followed closely by the e-helpers and clinical supervisor.

The dropout rates were reported separately for starters- users who started session 1 (776 users); and the non-starters- those who stopped right after sign-up before accessing session 1 content. Completion was defined as doing four sessions out of five (295 users).

The drop-out rate among starters was 62%; this rate decreased further with more engagement in the intervention. In fact, of the people that started session 1 (1084) only 52% (560) proceeded to session 2, whereas after starting session 2, the dropout reduces considerably. Notably, 44.2% of users were non-starters. Thus, the total dropout rate including the non-starters was 84.8%. The detailed dropout rates per session are reported in Supplementary material S5. Most (94%) people who enrolled in SbS requested e-helper support. Of this group, 59% requested telephone support. The completion rate was highest in the group that received call support (32.7%), while those who selected message support had a completion rate of 15.2%. Users who opted for no support had the lowest completion rate (2.9%). Most people preferred the Arabic version of the program (72%). Although most users (62%) were unmarried, 63% of all users picked the story line of the married character. Interviewees unanimously used the mobile application version. The overwhelming majority (>85%) of respondents to the satisfaction questionnaire indicated that they were at least “mostly satisfied” with the program. 89% indicated that they would go back again to this program if they required support in the future.

Qualitative findings revealed high satisfaction with SbS among users, whereby most intervention completers and even those who dropped out (except for one interviewee) had their expectations met or exceeded. The flexibility, user-friendliness, simplicity, and relevance of the program- particularly the narrative style and practical activities like the self-care, slow breathing, gratitude, and mood tracker- were mentioned as key enablers for adherence and uptake. One exception was the grounding exercise in the introduction which triggered negative reactions like distress and discomfort. The more complex activities were not commonly used due to time constraints, the planning required, and the complexity of the online process. Notably, the endorsement of SbS by some professional therapists motivated users who were already in therapy to adhere and report their progress to their therapists. Despite initial skepticism about the effectiveness of SbS among some, users rapidly grew trust in the program upon early contact with empathetic and professional e-helpers. Most users and e-helpers preferred personalized phone call support over messaging for conveying empathy and rapport-building. Nonetheless, messaging was helpful for those with privacy concerns or social anxiety. Barriers to adherence included usability issues (long assessments, technical bugs, poor internet quality) and user-related factors (emotional burden and exhaustion, demotivation, time management, competing priorities, forgetfulness, lack of understanding of the exercises and their purpose, and preference for specialized face-to-face support). Nonetheless, most users who were interviewed reported a sustained engagement with the program during and after completion. Frequency of use varied between daily, weekly, need-based usage (when feeling down or anxious), and routine-based (i.e., after putting child to sleep). Some of the early enrollees in the pilot reported sustained engagement even after 2 years (by the time of the data collection) and noted long-term impact of SbS whereby most still integrate the activities and skills in their daily lives beyond the program. “*A lot of people did disappear in session 2 or 3 [and] told me that they were still doing the activities, and still write what they are feeling*” (e-helper 1). Reported outcomes by users and staff include mood and emotional regulation, improved ability for self-care, and increased confidence and autonomy to implement the skills and manage distress and triggers. Recommendations from qualitative interviews with staff, users and partners revolved around considering an adaptive delivery model regarding the pace and content of the program, the frequency and length of support sessions, as well as enhancing the application’s engagement and nudging, and improving the technical aspect to reduce its bugginess.

### Adoption

The NMHP introduced SbS in MHPSS task force meetings and actively approached five NGOs, seven Primary Health Care (PHC) centers in South Lebanon, two universities and one private sector organization to adopt SbS.

The majority of the organizations (86.6%) that were approached signposted SbS to their beneficiaries. While most users learned about SbS through social media, only 4.8% learned about it from healthcare workers. By the end of the study, it was reported that SbS had not been formally and systematically integrated as part of the range of depression treatments offered in most of the facilities. Nonetheless, it was being introduced in the national trainings on the integration of mental health care packages in the PHC network. Qualitative interviews with disseminating organizations reported that most partners were confident and comfortable to disseminate SbS and valued its importance given the high need for mental health care and the short resources and funds allocated to the PHC network in Lebanon. Nonetheless, some barriers to adoption included concerns about the ability to manage users at high risk of suicide through a remotely delivered intervention, concerns about the e-helpers’ capacity to support a large caseload should the program be disseminated widely to their network, the high staff turnover rate and the need to train continuously on SbS in PHCs, as well as the inaccessibility of some vulnerable population groups to the internet or to technology, especially for NGOs and PHCs. A set of recommendations was generated by the stakeholders and project staff, including more rigorous monitoring of the integration process and referrals by organizations, meaningful engagement of stakeholders in the design and piloting of the scale up model, a structured and standardized integration approach of SbS into a national model of care in various facilities, and training of frontline staff to refer to SbS whenever relevant.

### Implementation

On average, the 295 users completed SbS within 46 days with 77.6% completing the program in 2 months or less, consistent with the five-to-eight-week delivery model. Support calls averaged 14 min in line with the e-helpers’ support model, except for calls with high-risk users which averaged 43 min. Fidelity checks conducted by the clinical supervisor and senior e-helper (176 fidelity checks, 31% related to phone calls and 69% related to messaging) revealed high compliance to the protocol and high acquisition of skills among e-helpers, with 93% compliant with the requirements set by the supervisor. The mix of the four-day initial training, the 1.5 months on the job practice, and the supervision meetings was praised by all staff. Few recommendations included providing staggered training sessions and a mentorship program for new staff- both changes to the protocol were subsequently adopted. Other protocol modifications and mitigations that took place during the pilot included moving to remote work during COVID-19 pandemic, shifting from volunteering to employment model during the economic crisis, and launching an internship program for university students to account for the staff turnover. Despite the 50% staff turnover rate at the beginning of the implementation due to COVID-19 concerns, economic instability, and lack of career advancement, retention was enhanced by the healthy work environment, strong peer and supervisory support, professional development, and the rewarding nature of the job “*It definitely shaped and will shape how I approach my role in the future as a therapist. The impact on people’s lives is beyond measurable*” (E-helper 1). Staff recommendations to improve implementation included continuous training, designing a career ladder, and implementing structural and staff-care initiatives to promote the well-being of the staff.

### Maintenance

Stakeholders’ discussions on sustaining SbS in Lebanon recognized its main strengths as it being evidence-based, free-of-charge, widely accessible, and supported by real humans, and weaknesses being inaccessibility to the internet or compatible phones among refugees and the costs of running an e-helper service. While some partners suggested a volunteering or internship model as a cost-effective solution, the overwhelming majority acknowledged the importance of reimbursing e-helpers for their demanding and sensitive job. Outsourcing of management and administration (logistics, IT, and procurement) to a local implementing partner was deemed beneficial by partners; yet a need for improvements was noted relating to communication of SbS as a national service by NMHP, division of roles, and coordination. Threats to sustainability included economic instability and funding shortages, deteriorating quality of life and infrastructure, political pressure on refugees to leave the country, and rapid technological growth. Partners suggested officially integrating SbS into the national mental health care pathway in primary care by MoPH. Recommendations included conducting national dissemination efforts in universities, orders, syndicates, mass and social media. Sustainability recommendations included upholding multisectoral coordination and securing long-term funds from the public and private sectors and research grants.

## Discussion

This implementation study evaluated the real-world performance of SbS in Lebanon, demonstrating its effectiveness, uptake, and public health benefits, while showcasing the barriers and enablers to its integration in the healthcare system across the RE-AIM domains. In terms of *Reach*, despite Lebanon’s compounded crises, contextual challenges and competing needs, as well as initial skepticism against digital interventions, the recruitment target was exceeded thus shedding light on the unmet mental health needs and the feasibility of digital interventions in volatile settings (44). The high satisfaction and perceived relevance of SbS prove that the participatory approach adopted in co-designing and culturally adapting SbS in Lebanon was effective, yet the completion rate of 38% among starters suggests that further improvements are needed to boost it (16). Social media proved to be a successful recruitment tool for Lebanese people but not to Syrians who constituted only 11% of participants. Hence, adopting various recruitment channels such as community outreach to Syrians might be needed. This is consistent with similar challenges observed in Egypt, Sweden, and Germany where outreach volunteers or trusted “champions” were essential in recruiting Syrians (12). Qualitative results implied that endorsement by mental health professionals might lead to better trust, improvement, and completion rates among users. Hence the importance of targeting psychologists and psychiatrists in orientation sessions around SbS and encouraging them to link their waitlist or current clients to this service.

As for *Effectiveness*, SbS maintained its effectiveness with large effect sizes in reducing depression and anxiety symptoms among users including those with severe symptoms at baseline (*d* = 0.91 and 0.96 respectively), and moderate effect size for functioning, *d* = 0.63. These results counter concerns about reduced effectiveness of digital mental health interventions upon scale-up (7, 45). Although effect sizes for one-group studies are not directly comparable to those of an RCT given the different designs and sources of variability, and the absence of control conditions, nonetheless, the findings suggest that evidence is converging toward the sustained effectiveness of SbS in real-world setting. As expected, our effects sizes were larger than those reported in the RCT, consistent with evidence that uncontrolled studies tend to overestimate the treatment effects as they do not control for external factors, natural recovery, and other aspects (4, 5, 46). Nonetheless, this study demonstrates that SbS continues to achieve clinically meaningful results in treating depression and anxiety in routine care settings. The 41% response to treatment for depression in this study, which is defined as a reduction of more than 50% in PHQ-9 scores from baseline to post-test, aligns with the rates observed in the SbS RCTs of 46.5% for Lebanese, and with the overall response rates observed in meta-analysis for psychotherapies for depression symptoms at 2 months post-baseline (41%) (47). The remission rate of 18.6%, defined as scoring below 5 on the PHQ-9 at post-test, is lower than observed in the RCT for Lebanese in the intervention group (26%), yet still demonstrates clinically meaningful improvement (4, 5). Further analyses are needed to compare the effectiveness in the two different settings, yet SbS appears to maintain its clinical relevance and benefit in the real-world setting despite contextual differences, thus supporting the scale-up and public health benefit of SbS in Lebanon. Notably, preliminary analyses showed that people with more severe depressive symptoms benefited more than those with lower-level depression at baseline. This is congruent with existing evidence that non-specialist delivered psychological interventions show greater benefits among those with more pronounced problems at baseline (48). This finding suggests that SbS could complement existing mental health interventions, including stepped care, and provide access to care for those who exhibit severe depressive symptoms and lack access to specialized services, especially across large geographical areas. High satisfaction with SbS was linked to its simplicity, practicality, relevance, storytelling approach, and empathetic support, in line with the literature (16, 49–51). Nonetheless, privacy concerns persisted as shown in other studies, revealing the need to continuously reassure users and uphold data security and privacy practices (52, 53). While the dropout rate was considerably high (62% among starters), it is relatively comparable to the 60% dropout rate reported during the Lebanon RCT among intervention starters where follow up and compensations were used (5). Attrition mainly occurred prior to initiating the sessions of the program (40%), particularly among those who opted for message support. This is comparable to the non-starter rates of SbS research in China and the feasibility study in Lebanon (36.8, 42.6% respectively), indicating the need to engage with users at early phases (54, 55). It is noteworthy that digital mental health interventions that offer human support consistently show higher adherence rates than unguided interventions, as shown in a systematic review whereby 47% of participants completed all modules in guided interventions compared to 29% in unguided ones (8). Qualitative studies have demonstrated a strong preference among users for human guidance to complete the interventions and acknowledged the need to introduce guidance, therapeutic alliance, and follow-up to retain participants (9). Reviews have even recommended that human support in guided interventions becomes mandatory rather than optional to improve adherence (8). Common reasons for dropout and enablers to adherence captured in this study are consistent with the literature and suggest that the intervention usability, length, e-helper guidance, and personal factors are all equally crucial factors in promoting or hindering adherence of the users to the SbS program (56). Human support and the introduction of a welcome call for all users, mid-implementation, were highly praised by users and have started to show a shift in adherence patterns according to the e-helpers. Although high drop-out rates are common in e-mental health interventions, they do not necessarily signal no public health benefit. Qualitative results showed that some users who did not complete the program reported integrating the skills and the simple techniques into their daily lives over the long run, with quantitative results showing that completion led to clinically meaningful changes. Although frequent engagement with digital mental health applications is linked to better clinical outcomes (57, 58), yet it is common for users of digital mental health interventions to engage less frequently (9). Several studies have shown that minimal engagement with such applications can still have a positive effect on clinical mental health outcomes (9, 57), so in the current study the impact of SbS on non-completers is unfortunately, unknown. However, the results suggest that for completers and perhaps some non-completers, there is a great potential for digital interventions to have a lasting positive impact on people’s mental wellbeing.

*Adoption* of SbS in Lebanon and integration in the existing system is still in progress. Organizational readiness to adopt varied considerably; while some fully integrated SbS into their referral protocols, others took longer to adopt it or only posted informational material in their centers. Universities and NGOs acknowledged the need for such interventions amidst the shortage of mental health professionals, yet highlighted the need for more staff training, a standardized referral model and effort to fit it into legal and technological infrastructures to completely integrate SbS in the system. Although 86.6% of partner organizations, including PHCs, agreed to promote SbS to their beneficiaries, only 4.8% of users reported being referred to by healthcare workers. This highlights a critical gap. While PHCs are among the most accessible entry points to mental health services in Lebanon, low familiarity with SbS among PHC staff might have limited its referral. Although SbS is now formally included in national mental health referral pathways and information materials, nonetheless, effective integration at the PHC level requires more than dissemination; it necessitates practical training, first-hand engagement with the tool, and sustained follow-up mechanisms within PHCs.

Under *Implementation* assessment, this evaluation proved that it was feasible to implement SbS as intended amidst a highly volatile setting. Mitigation measures such as shifting to e-helper employment model, remote work flexibility, and robust supervision were crucial to counter barriers such as power and internet outages, road blockages, COVID-19, and staff turnover. Risk of turnover among e-helpers was high yet staff retention was driven by intrinsic motivation, supportive leadership, and professional development, supported by literature (59, 60). The capacity building, supervision, and remote delivery model proved to be effective in identifying and supporting individuals at high risk of suicide, SGBV, and child protection.

As for *Maintenance*, the best practices for the scale up of SbS in Lebanon included governmental endorsement and ownership, building robust and sustainable partnerships with local and international agencies, and an employment-based model of e-helpers. Common challenges to scaling-up entailed technological limitations and funding scarcity, both common to digital interventions in low-resource settings (61). A study assessing scalability of SbS in Egypt, Sweden, and Germany unveiled the complexity of integrating it into an existing system and the required preparedness at the legal, structural, and organizational levels to sustain the program (12). As global interest grows in scaling up interventions like SbS for different population groups, integrating it into the primary health care network in Lebanon as part of the national mental health referral model by the NMHP at the MoPH presents one cost-effective response to help address the mental health treatment gap. SbS is already being integrated in the trainings of healthcare workers on available resources in the country. SbS could also be featured on the platforms of all national services developed by NMHP and partners such as the 4Ws platform which maps MHPSS services in Lebanon, the Self-Help Plus and the Mental Health in the Workplace platform that are under development, and any awareness material around national resources for mental health. However, threats such as political pressures on Syrians to leave the country, high dropout, and the fast technological advancement must be tackled. It is thus imperative to invest in technical adaptability of digital interventions in LMIC to ensure their viability. Overall, to move from mere dissemination to full integration, multisectoral long-term partnerships are needed to secure sustainable funding, conducive infrastructure, adaptive technology and a wide dissemination and adoption of SbS in Lebanon.

### Study limitations

The main limitation of this study was the difficulty to reach people who dropped out to garner more feedback and recommendations as they were not very responsive to the survey and to the key informant interviews invitations. Another limitation was the possible bias generated by interviewing stakeholders affiliated with SbS and NMHP. Finally, the discrepancy of interview methods for SbS staff (face-to-face) and SbS participants and external stakeholders (phone) can be considered as a limitation to the methodology.

### What this study adds

The public health impact, effectiveness, and uptake of digital mental health interventions in real-life setting beyond the research phase is under-researched in LMICs among vulnerable groups and populations affected by adversities. This implementation pilot evaluation helps understand the performance of such interventions in similar settings and identifies challenges, best practices, opportunities, and threats to consider for further scale up. This pilot provides critical insights to inform the decision for a full scale-up of SbS in Lebanon and highlights key criteria for the sustainability of this model in Lebanon. Following this study, we recommend conducting a focused case-study within a primary care center to assess the incorporation of SbS into a comprehensive stepped care model. Moreover, this study supports the feasibility of the RE-AIM framework in evaluating mental health interventions’ implementations in LMIC. Policy makers and donors could draw on these findings to guide their decision to invest in digital mental health interventions and to decide on the scale-up model relevant to their settings.

## Conclusion

SbS has demonstrated effectiveness and public health impact in treating depression and anxiety among people living in Lebanon, a lower-middle-income country with a challenging context, outside of a research setting. These findings support the feasibility and scalability of SbS in the real world as a solution to increasing accessibility to mental health care among people living in adversity. Despite the high dropout rates and uptake challenges which require further in-app improvements and user engagement strategies, SbS presents a cost-effective solution to the shortage of mental health professionals in Lebanon by providing accessible evidence-based treatment under the supervision of one clinical psychologist and a team centralized in one place. While integration of SbS into the existing system has not fully taken place, a follow up case-study and a hybrid effectiveness-implementation study within primary care centers would be crucial to assess its incorporation into a comprehensive stepped care model and identify the best integration model. This study highlights the importance of adopting a system strengthening approach while investing in new interventions to ensure their proper embedding into the various levels of care. SbS has attracted international recognition, including the 2023 UN Interagency Task Force Award (62), which reflects global interest in upscaling digital mental health interventions and sustaining them, presenting an opportunity for a full-fledged integration into the healthcare system in Lebanon, and securing sustainable funds through multisectoral, local and international partnerships.

## Data Availability

The raw quantitative data supporting the conclusions of this article will be made available by the authors, without undue reservation. The interview transcripts are not readily available for confidentiality purposes.
