# Supplementary material for: From research to real-life implementation: an evaluation of the scale up of a guided digital mental health intervention in Lebanon: Step-by-Step
Source: Front Public Health. 2025 Nov 11;13:1665093. doi: 10.3389/fpubh.2025.1665093 (PMC12643871; doi:10.3389/fpubh.2025.1665093)
Supplement: Supplementary file 1 [file Data_Sheet_1.DOCX]

This Informed Consent Form is for project participants, staff and stakeholders who we are inviting to participate in qualitative research on the evaluation of an internet-based health programme. The title of our research project is Step-by-Step.

**Site principal investigator: Dr Rabih El Chammay, National Mental Health Programme, Lebanon.**

**This Informed Consent Form has two parts:**

- **Information Sheet (to share information about the research with you)**
- **Certificate of Consent (for oral confirmation if you agree to take part)**

**You will be given a copy of the full Informed Consent Form**

**PART I: Information Sheet**

**Introduction**

The “Step-by-Step” intervention is an evidence-based, innovative approach to treatment for depression using a five-session internet-delivered self-help intervention for adults experiencing common mental health issues. The World Health Organization (WHO) and the Ministry of Public Health (MoPH) tested its feasibility and effectiveness through a Randomized Controlled Trial (RCT) in Lebanon and now it is available as a service. People interested in this program can access Step-by-Step through their own device (app or web-browser). “Step by Step” users will receive technical and motivational support each week from e-helpers, who are non-specialists trained to provide basic support for the users. The National Mental Health Programme (NMHP) at MoPH has scaled up the step-by-step (SbS) program in to a free national service. We would like to get your feedback and insight on the implementation of such e-mental health program in Lebanon.

**Purpose of this evaluation**

As you know, we have designed a programme to help people living in this area who are dealing with difficult emotions. Difficult emotions, such as feelings of sadness or stress are very common, especially when people are struggling with problems in their lives.

Now that you’ve been involved in planning, disseminating, delivering or using our programme, we believe that you can help us by telling us your thoughts about the programme, both good and bad. We would like you to be honest about the programme, in order to help us to improve it.

**Type of Research Intervention**

This research will involve your participation in one interview that will take about 45 minutes.

**Participant Selection**

You are being invited to take part in this research because you have had experience with the programme in some way and your experience can help us to make the programme better.

- ***Question to check understanding:*** *Do you know why we are asking you to take part in this research?*

**Voluntary Participation and Right to Withdraw**

Your participation in this research is voluntary: That means that it is your choice whether or not to be in this research project. No one is forcing you. You may change your mind at any time. You can stop the interview at any time, and you can also ask that your answers are not included in the research. Deciding not to participate, or stopping the interview, will not have any negative impact on you or your work.

At the end of the interview, if there is something that you said that you wish to not be included in the research, you can tell us and we will leave it out. If there is something you want to change about what you said, you can also tell us that, and we will change it in our notes.

***Questions to check understanding:*** *Do you know that you do not have to take part in this research project, if you do not wish to?*

**Procedures**

If you agree, we will ask you some questions about your experiences with the programme.

We will ask you to have an interview with an interviewer from the Ministry of Public Health Lebanon. The interviewer will call you, it’s recommended to have the call in a private and comfortable place where you won’t be disturbed. If you do not wish to answer any of the questions during the interview, you may say so and the interviewer will move on to the next question. The interviewer will not be upset if you have any negative comments about the programme.

No one else but the interviewer will be on the call. The interviewer will use a recorder to record the interview, so that we can listen again later to what you told us. We will keep this recording safe. The interview will last for about 45 minutes. If we need some more time to ask all of the questions, or if you have more information to give us, the interviewer will arrange another call.

***Questions to check understanding :*** *If you decide to take part in the study, do you understand what would be involved? Do you know that you may not answer the questions that you do not wish to respond to?*

**Risks**

If you find any of the questions too personal, uncomfortable or difficult to answer you do not have to answer them.

You do not have to give us any reason for not answering any question, or for not completing the interview. If you become very upset from talking about your experience of the programme, you will be able to speak to a member of staff who will support you.

**Benefits**

There may not be any benefits to you from completing this interview, but your participation is likely to help us to develop a more useful programme to help local people in managing emotional problems.

**Reimbursements**

You will not receive any compensation for participating in the interview research.

- ***Questions to check understanding:*** *Can you tell me if you have understood correctly the benefits that you will have if you take part in the interview?*

**Confidentiality**

## All researchers working on this project will ensure privacy and confidentiality for all information and documents from the research.

## All information collected about you will be kept strictly confidential. What we record on the recorder will be listened to and written down, and we will not write down any names. Once it is written down, we will erase this recording.

## Any information about you will have a number on it instead of your name, and a list linking your name to your number will be kept safe. Data will be stored on a computer and only the research team will have access to it. Any results that we report will not reveal the identity of any participants.

The data will be destroyed after 10 years post-research.

- ***Questions to check understanding:*** *Do you understand how we will make sure that any information that we as collect about you will remain confidential? Do you have any questions about this?*

**Sharing the Results**

The knowledge that we gain during this research will be shared in a general only. We will publish what we have learnt so that other interested people may learn from this research, but we will never include information which would identify you as having taken part. The information we gain will be used to further develop the programme.

**Who to Contact**

If you have questions now you can ask me. We will also give you the name and phone number of a study team member to contact if you have questions later. This person is: Dr Rabih El Chammay, Ministry of Public Health, Phone no: 01611672, ext: 125.

This proposal has been reviewed and approved by the Institution Review Board of the Saint Joseph University, Lebanon. This is a group of people whose task it is to make sure that research participants are protected from harm. If you wish to find about more about the committee, contact Dr Rabih El Chammay, Ministry of Public Health, on 01611672, ext: 125.

***Questions to check understanding:*** *Do you know how we will share the results of the study?*

***Question:*** *You can ask me now, or call or email us later to ask any questions about any part of the research study, if you wish to. Do you have any questions?*

**Part II: Certificate of Consent**

I have been invited to participate in research about emotional problems in our community.

I have read and understood the above information, or it has been read to me. I have had the opportunity to ask questions and any questions I have been asked have been answered to my satisfaction. I consent voluntarily to be a participant in this study.

**Print Name of Participant__________________________________________**

**Signature of Participant ___________________________________________**

**Date ___________________________**

**Day/month/year**
