# Supplementary material for: From research to real-life implementation: an evaluation of the scale up of a guided digital mental health intervention in Lebanon: Step-by-Step
Source: Front Public Health. 2025 Nov 11;13:1665093. doi: 10.3389/fpubh.2025.1665093 (PMC12643871; doi:10.3389/fpubh.2025.1665093)
Supplement: Supplementary file 2 [file Data_Sheet_2.DOCX]

**Increasing Access to Mental Health Care for People in Lebanon Living in Adversity:**

**“Step-by-Step” intervention- Stakeholders guide.**

**Add intro about SBS:**

The “Step-by-Step” intervention is an evidence-based, innovative approach to treatment for depression using a five-session internet-delivered self-help intervention for adults experiencing common mental health issues. The World Health Organization (WHO) and the Ministry of Public Health (MoPH) tested its feasibility and effectiveness through a Randomized Controlled Trial (RCT) in Lebanon and now it is available as a service. People interested in this program can access Step-by-Step through their own device (app or web-browser). “Step by Step” users will receive technical and motivational support each week from e-helpers, who are non-specialists trained to provide basic support for the users. The National Mental Health Programme (NMHP) at MoPH has scaled up the step-by-step (SbS) program in to a free national service. We would like to get your feedback and insight on the implementation of such e-mental health program in Lebanon.

**Qualitative Evaluation Interview Guide**

**This section is for all stakeholders:**

1. **General feedback**
   1. Based on your understanding of SbS (self-help app with guidance from a trained helper), are you satisfied / supportive of the model and approach taken?
   2. What were the feedback/impressions you heard about SBS? From whom?
2. **Feasibility, acceptability and relevance**
3. To what extent do you think this service is acceptable among the Lebanese, and among different populations residing in Lebanon (Syrians, Palestinians)?
   - 1. Are there any population groups it would be more or less feasible, acceptable or relevant to? (age, nationality, literacy, etc…)
     2. What do you recommend to maximize acceptability, relevance, and feasibility of the app for different population groups? Do you have any suggestion related to new relevant target groups, new stories (e.g. refugees or migrant workers etc.)?
4. Are there any changes you would suggest that might increase its feasibility, acceptability or relevance when scaled up on the long run?

**This section is for partners only (NMHP, WHO, Embrace) :**

1. **Implementation and project placement in Lebanon - Assessment of Partnership on SbS:**
2. What was your role in the project? And the role of other partners (between NMHP, WHO, and Embrace)?
   1. Did the degree of involvement of each partner meet the original expectation of each organization?
3. In general, what were the strengths, weaknesses, threats and opportunities for the project and this partnership between Embrace and NMHP? What worked well? And what didn’t?
   1. Was there enough transparency? Did a delay in a partner activity cause delays in other partner’s activities or action plan?

**If these were not covered in question b, please proceed with the detailed questions below:**

1. What is your overall impression of the communication and coordination between the hosting partner and the NMHP?
   1. Are there any challenges encountered with communication and coordination between the two parties?
   2. What were the strengths encountered with communication and coordination between the two parties?
   3. What would you change about the communication and coordination model between the two parties?
2. What is your overall impression of the hosting partner’s role in supporting SbS through their various functions? Positives, negatives, recommendations of each of the below functions:

Were the interventions of different actors harmonized and complementary, rather than duplicated? Was there enough level of coordination between the members at an operational level? What can be done to improve the coordination?

- 1. Human resources management and maintaining a service delivery model i.e., recruiting, training, provision of admin support.
  2. Quality assurance, clinical supervision, performance management i.e., attending monthly group supervision meetings, following up on the progress of the project.
  3. Managing finances and logistics i.e., handling budget, expenses, finances, procurement of project needs (phones, recharge cards, computers, internet connection, other materials, office space, etc.)
  4. Ensuring participants’ safety and support i.e., handling imminent and extreme cases.
  5. Supporting in programming and technical needs i.e., IT and technical issues.
  6. Supporting in communication and dissemination strategy i.e., admin support and overall support, revision and advice on social media post and sharing social media posts on their pages, outreach events, liaising with partners.

1. Were there any changes implemented in the work plan? (i.e., fidelity to original work plan/TOR) to what extent have the lessons learnt from the research phase been taken into account and followed during the implementation?
2. What other challenges were encountered during the implementation phase especially for the staff (e-helpers, supervisor, and coordinator) ?
   - 1. What were some mitigation measures that helped overcome those challenges ?
3. If this partnership is to continue, what are the things that need to be changed?
   1. What can be done to improve the partnership and better meet the needs of the project and its users?

**This section is for all stakeholders:**

1. Two challenges we faced during the implementation study were recruitment (ensuring people knew about the service and its availability), ensuring people did not drop out. Do you have any suggestions on how we might address these challenges? What other challenges do you foresee in the future scale up of SBS in Lebanon? How do you think can we tackle/avoid those challenges? (cost, retention of e-helpers, retention of users, lack of incentives for users, etc..)
2. Who are the stakeholders that we would need to involve in order to run SbS or to ensure SbS is well disseminated amongst networks? (public, private sector, NGOs, syndicates, etc…). What would the role of NMHP be after the implementation study? That of WHO? And Embrace?
   - 1. What is your overall impression on involving you as hosting partner to run SbS and ensure its dissemination? (i.e., is it required? etc.)
     2. Do you believe SbS requires the involvement of more and/or different stakeholders to keep it running and maintained? If so, what kind of stakeholders do you believe would need to be involved?
3. What are the strengths, weaknesses, opportunities, and threats for Step-by-Step in the future? What are some facilitators and available resources that can support the implementation of this program? (Context-related, external, internal, funds, resources, dissemination, acceptability)
4. What recommendations do you suggest for a better implementation of the service and higher effectiveness and reach?
5. What is your assessment of the cost of this project overall? (with the changes made in the upscale). How do you foresee the funding of this project to happen on the long run? What are some challenges foreseen? What are possible funding schemes (what would be the role of WHO Lebanon, Embrace)?
