# Supplementary material for: From research to real-life implementation: an evaluation of the scale up of a guided digital mental health intervention in Lebanon: Step-by-Step
Source: Front Public Health. 2025 Nov 11;13:1665093. doi: 10.3389/fpubh.2025.1665093 (PMC12643871; doi:10.3389/fpubh.2025.1665093)
Supplement: Supplementary file 3 [file Data_Sheet_3.DOCX]

Interview Guide: E-Helper

Greet person. Introduce self, including what organization you are working for. Explain the study, following written informed consent process.

Possible additional explanation of semi-structured interview process:

*You have recently participated as an e-helper in the Step-by-Step implementation study. We would like to ask you some questions about your experience with the service and the platform, to help us to think about how Step-by-Step and the procedures could be improved for delivery in the future.*

*There are no right or wrong answers to the questions we are going to ask. The interview will be recorded without any identifier to make sure that answers are transcribed accurately; all answers will be reported anonymously to ensure confidentiality. We will be speaking to a number of people, asking everyone the same questions. If you feel unable to answer a question please say and we will move on to the next one.*

In note book document date and site of interview, age and gender of interviewee, who they are (i.e. intervention participant, helper, policy maker), and initials of interviewers.

Begin semi-structured interview:

Record responses and take down pertinent responses in the notebook.

Interview process:

**Overall impressions:**

1- How was your experience in taking part of a nationally delivered electronic self-help program SBS? Explore positive / negative views through probes.

2- Do you believe that an electronic guided self-help service can be useful/accepted in Lebanon?

**Intervention:**

*Content, features and delivery method*

3-Please think back to the users’ experience of using the website/app and reflect on their experience using the app, including features and design. (How was it for them to navigate through the sessions, exercises, did they like the colors, etc..)

1. What are the features/exercises they seemed to like the most?
2. What are the features/exercises they seemed to not like and why?
3. Which features were mostly used by the users and which were not used at all?

4- What alternative features or options do you recommend to be present to help improve users’ experience?

5-Did you experience any differences between messaging and call support? Which was more effective and comfotable to conduct? Which method can better improve the user’s experience in the program? What changes can be implemented (Adding more calls per week, longer calls? Chatting versus messaging?)

**Experience with clients:**

6- Can you describe your rapport with clients you have supported?

1. Explore positive / negative views through probes. (If they are not forthcoming to open questions, can ask about the following: follow up with users, relationship, most encountered challenges, difficulties, positive changes)
2. What was your role in ensuring that the users experienced a safe space while using the app?

7-Did you encounter any difficulties with families of clients you have supported or suspected any risk issues arising from communications with your clients?

1. Explore positive / negative views through probes

8-Do you think users will be able to use Step-by-Step without the e-helpers support at some point?

**Intervention adherence:**

9- Based upon your experience, what would help people stay motivated to engage with Step-by-Step and practice or use some of the techniques.

10- What were some common reasons for dropout or unresponsiveness?

1. What could help/helped in improving the adherence?

11- How much do you believe that SbS played a role in improving users’ depression and well-being? Did you note any improvements in the users’ mental states throughout the exercises, or through the discussions that took place between you and the users?

13-To what extent do you feel your clients implemented the skills they learned in the program? Explore barriers and facilitators to skills development and skill application into their daily life.

**Local management and supervision:**

14- What is your feedback on your shift and time/workload management during the shift?

Caseload – what do you think is a manageable number per day / shift?

Is the 15 mins reasonable? Did it take you some time for that to feel ok, if so, how can we support with that?

Is there anything that was not necessary or that needed to be amended? (case notes/etc..)

15- What is your overall impression on the supervision model (weekly meetings/face to face)

- - - - 1. How do you describe its effectiveness in solving problems encountered? (Were you able to discuss and solve challenges in your team meetings? What could be improved?

16- What would you add or change about the helper manual and other materials (SOPs, etc..)? is there anything that you wish was there? More guides, tips, processes, templates.

17- Based on your experience, how was the orientation phase including training and support provided during the initiation phase? Was the training length and structure acceptable? (3 days theory about research and intervention and one-month practice).

18-Do you recommend any changes? is it sufficient and clear to start the implementation phase directly or is there a need for further training? (Would you suggest any changes?)

19- What do you need to get comfortable in your role? Looking back, anything you would have liked to know at the start?

20- What would help you to stay in this job? and what are some possible reasons for you to leave this job?

1. What do you think you need to grow in your position? (e.g., any trainings or capacity building workshops)

22- How did this job affect you?

1. Positives/negatives/stressors
2. How did this affect your wellbeing?
3. Recommendations to minimize the stressors/negatives and maximize the benefits/positives and prevent burnout?

### **Assessment of Partnership with Hosting Partner (Embrace)**

23-What is your overall impression with the hosting partner?? (i.e., support etc.)

1. In your opinion who are the stakeholders that we would need to involve in order to run SbS or to ensure SbS is well disseminated amongst networks? (public, private sector, NGOs, syndicates, etc…). What would the role of NMHP be after the implementation study?

Review any written records with the interviewee still present. If anything is not clear, ask for clarification and correct written notes as necessary.

Ask the interviewee if they have anything to add. Any additional information is added to the interview notes as required.

Thank person and leave.

Interview Guide: Clinical/admin supervisor

Greet person. Introduce self, including what organization you are working for. Explain the study, following written informed consent process.

Possible additional explanation of semi-structured interview process:

*You have recently participated as clinical supervisor in the Step-by-Step implementation study. We would like to ask you some questions about your experience with the programme and the service, to help us to think about how Step-by-Step and the procedures could be improved for delivery in the future.*

*There are no right or wrong answers to the questions we are going to ask. The interview will be recorded to make sure that answers are transcribed accurately; all answers will be reported anonymously to ensure confidentiality. We will be speaking to a number of people, asking everyone the same questions. If you feel unable to answer a question please say and we will move on to the next one.*

In note book document date and site of interview, age and gender of interviewee, position and initials of interviewer.

Begin semi-structured interview:

Record responses and write pertinent responses in the notebook.

Interview process:

**Overall impressions:**

1- How was your experience in taking part of a nationally delivered electronic self-help program SBS? Explore positive / negative views through probes

2- How much do you believe that an electronic self-help service can be useful/accepted in Lebanon?

**User’s adherence:**

3- What were some common reasons for dropout or unresponsiveness?

4- What helped in improving the adherence to the app, exercise and e-helper support?

5- Are there any changes required to improve the experience in this program? (adding more calls per week, longer calls? Chatting versus messaging?)

6- Do you think users will be able to use Step-by-Step without the e-helpers support at some point?

**Helper retention:**

7- What are the motivators for the e-helpers to remain in their job? What are some possible reasons for you to leave this job?

8- In your opinion, how did this job affect the e-helpers?

- 1. Positives/negatives/stressors
  2. How did this affect their wellbeing?
  3. Recommendations to minimize the stressors/negatives and maximize the benefits/positives and prevent burnout?

**Integrating the role of supervisor into your workload:**

9- How do you view your role as clinical/admin supervisor to the e-mental health intervention considering your workload? Do you feel you need to allocate more or less time?

1. Explore barriers and facilitators to integrating the helping role into previous work.

10- What is your overall impression on the supervision model (weekly meetings/face to face) how do you describe its effectiveness in solving problems encountered? (were you able to discuss and solve challenges in your team meetings?)

11- Any other recommendations regarding the support of the e-helpers to solve the challenges? i.e. improving the way of supporting, the time per week, the individual discussions, ect.

12- Any recommendation to improve the coordination process between all project team members?

**Training needs of helpers:**

13- Based upon your experience, what would you suggest we change about the way the training is delivered? (e.g. language of training, length, role plays, classroom set up?)

14- Based on your experience, what would you add or change about the helper manual and training curriculum?

15- What would you add or change about the helper manual and other materials (SOPs, etc..)? is there anything that you wish was there? More guides, tips, processes, templates?

16- Based on your experience, how was the orientation phase including training and support provided during the initiation phase? Was the training length and structure acceptable? (3 days theory about research and intervention and one month practice).

1. Do you recommend any changes? is it sufficient and clear to start the implementation phase directly or is there a need for further training? (Would you suggest any changes?)

17- To what extent do you feel the e-helpers implemented the skills that they were trained in and that you taught them after they started their roles?

1. Explore barriers and facilitators to skills development

18- In your opinion, are the protocols suitable for all potential participants? What needs to be improved? (safety protocols etc…)

### **Assessment of Partnership with Hosting partner (Embrace) – This section is only for admin supervisor**

1. What is your overall impression of the communication and coordination between the hosting partner’s focal persons and the NMHP project coordinator on various functions?
   1. (Focal people: budgeting, HR, etc)
   2. Are there any challenges encountered with communication and coordination between the two parties?
   3. What were the strengths encountered with communication and coordination between the two parties?
   4. What would you change about the communication and coordination model between the two parties?
2. What is your overall impression of the hosting partner’s role in supporting SbS through their various functions? Please discuss positives, negatives, and recommendations of each of the below.
   1. Human resources management and maintaining a service delivery model i.e., recruiting, training, provision of admin support.
   2. Quality assurance, clinical supervision, performance management i.e., attending monthly group supervision meetings, following up on the progress of the project.
   3. Managing finances and logistics i.e., handling budget, expenses, finances, procurement of project needs (phones, recharge cards, computers, internet connection, other materials, office space, etc.)
   4. Ensuring participants’ safety and support i.e., handling imminent and extreme cases.
   5. Supporting in programming and technical needs i.e., IT and technical issues.
   6. Supporting in communication and dissemination strategy i.e., admin support and overall support, revision and advice on social media post and sharing social media posts on their pages, outreach events, liaising with partners.
3. Were there any changes implemented in the work plan? (i.e., fidelity to original work plan/TOR)
4. What is your overall impression on involving a hosting partner to run SbS and ensure its dissemination? (i.e., is it required? etc.) (This is the only question that will be asked to the clinical supervisor as well)
5. Do you believe SbS requires the involvement of more and/or different stakeholders to keep it running and maintained? If so, what kind of stakeholders do you believe would need to be involved?
   1. Who are the stakeholders that we would need to involve in order to run SbS or to ensure SbS is well disseminated amongst networks? (public, private sector, NGOs, syndicates, etc…). What would the role of NMHP be after the implementation study?

Review any written records with the interviewee still present. If anything is not clear ask for clarification and correct written notes as necessary.

Ask the interviewee if they have anything to add. Any additional information is added to the interview notes as required.

Thank person and leave.
