# Supplementary material for: From research to real-life implementation: an evaluation of the scale up of a guided digital mental health intervention in Lebanon: Step-by-Step
Source: Front Public Health. 2025 Nov 11;13:1665093. doi: 10.3389/fpubh.2025.1665093 (PMC12643871; doi:10.3389/fpubh.2025.1665093)
Supplement: Supplementary file 4 [file Data_Sheet_4.DOCX]

**Part 1. Interview Guide: Intervention Participants**

Greet person. Introduce self, including what organization you are working for. Explain the study following written informed consent process (Consent form at the end of this document).

Possible additional explanation of semi-structured interview process:

*We would like to ask you some questions about your experience in Step-by-Step, to help us to think about how it could be improved in the future. There are no right or wrong answers to the questions we are going to ask. The interview will be recorded without any identifier to make sure that answers are transcribed accurately; all answers will be reported anonymously to ensure confidentiality. We will be speaking to a number of people, asking everyone the same questions. If you feel unable to answer a question please say and we will move on to the next one.*

In note book document date and site of interview, age and gender of interviewee, their position but not their name (i.e. intervention participant, e-helper), and initials of interviewers.

Begin semi-structured interview:

Record responses and make pertinent notes in the notebook.

Interview process:

**Overall impressions:**

1-Please describe your experience in Step-by-Step. How do you feel about taking part of an e-mental health intervention?

1. Explore positive / negative views through probes.
2. Did you complete it? Or stopped? When?

2- To what extent did it meet your expectations? What were you hoping to get out of it?

3- How was your experience in being part of a digital self-help program?

1. What did you find good or not so good about it?

4- Have you experienced any technical issues or other issues using SbS? If yes get a short description using the probes:

1. Can you describe to me the problem overall.

5- To what extent did Step-by-Step fit your values’ system and cultural and personal needs?

1. Did you have concerns regarding confidentiality and privacy while participating in the program? To what extent do you believe that confidentiality and privacy of your shared information were maintained while you were using the app and interacting with the e-helpers?
2. How relatable were the story, characters, and activities, and how much were they fit to your culture?

6- Did you disclose to others that you were using the app?

If yes, how did you perceive others’ reactions and opinions towards your participation?

1. How did your family view the calls you received from your e-helpers or your relationship with them?
2. Explore positive / negative views through probes.

7- How did you know about SbS? Through a friend, social media etc,..

**Intervention:**

*Content, features and delivery method*

8- Can you tell me about your experience in signing up to Step-by-Step? (how was it for you to navigate through the questionnaires, study information, consent, did you like the colors, etc..)

1. Did you use the app or the website?
2. Did you use the app offline?

9- Can you tell me a bit about how often you used SbS and how many sessions you completed? What was your overall impression of it?

10-Can you think back to your experience of using the website/app and ask about experience using the app, including features and design. (how was it for you to navigate through the sessions, exercises, did you like the colors, etc..)

1. What are the features/exercises you used and liked the most?
2. What are the features/exercises you didn’t use and why?

Was there any point in the intervention that you remember the exercises being more complicated or too difficult, or *less helpful?*

What about the challenging activities?

11- For the each of the below activities, what did you think about them? What did you like or dislike about them? Which ones did you mostly use, and which you did not, and why? What do you think about the logos and icons? Are they meaningful?

- Grounding exercise
- Breathing exercise
- Gratitude list
- Small activity
- Challenging activity
- Problems and Solutions
- Kind to yourself activity
- Social activity
- Warning signs
- What alternative features or options do you recommend to have? To help improve your experience?
- Probe: is there anything you would add? Anything you would remove?

12-What did you think of the story? (probe if needed: how did you feel about its relevance, redundancy, difficulty, engaging and captivating...)

13-Did you download the audios to listen to the story or did you prefer to read it/calendar?

1. What did you think of the audio exercises? (probe if needed: grounding and relaxation; idea, difficulty, effectiveness voice, pace, placement in the app)
2. What could be improved?

14-How did you feel about the 4-days wait between the sessions? Can you reflect on this experience?

1. Was there any preferred session? Or a session you didn’t like? Why?

*Outcomes [improvement in depression and well-being]*

15- Following your participation in the SbS program, have you experienced a change in your wellbeing?

1. First as immediately after participation and then a few months after they finished from the participation
2. Was there a relapse?

16- Are you using / have you used any of the learnings from SbS, in your daily life?

1. If yes, what are you using? If no, what’s got in the way?
2. How confident are you in performing the acquired techniques and coping mechanisms in managing distress and problems that arise in your daily life or that you experienced relapse post participation in the SbS?
3. What are some coping mechanisms you are using in the current situation? (before Covid, after Covid and after explosion, socioeconomic situation) And are the examples provided in the app relevant to the current stressors? (covid, explosion, socioeconomic situation and instability)
4. How we can make it easier for people to use the techniques in their daily life or come back for more guidance to the program?

17. What can be done to help users benefit from the app (videos, reminders, interactions)

**Rapport with helper:**

18- Please describe how you found working with your helper

1. Explore positive / negative views through probes. (technical, motivational, emotional)
2. Did you experience a safe space while participating in the SbS program? What was the role of the e-helpers in contributing to this? What did the e-helpers do to ensure your privacy and confidentiality and gain your trust?
3. If you are using calls, how did you find the e-helper’s tone and approachability, level of empathy? Do you think you were able to connect with the e-helper?
4. If you are using messaging support, how did you find the messages being sent to you? Did you think the e-helper was empathetic? What do you think about the length of the messages? Were they repetitive?
5. How comfortable are you disclosing sensitive topics through the both means of contact?

19- Do you think the app would function well without the e-helpers? Why?

**Intervention adherence:**

20- For people who dropped out:

1. What were your reasons for dropping out of the app?
2. Is there anything that would have helped/motivated you to stay in the app?

21- Please describe how easy or difficult you found it to complete the five sessions

1. Explore barriers and facilitators to completing the intervention (frequency of sessions, length, etc…)

22-Is there anything else we could do to help users stay motivated, keep using the app and responding to e-helper support?

1. Explore further if they are not clear.

**Recommendations for new features:**

23- What additional or new features you would like to see or use if you had the chance to re-participate in a new version of the Step-by-Step program?

24-Would you recommend others to use the app? Why?

Review any written records with the interviewee still present. If anything is not clear ask for clarification and correct written notes as necessary.

Ask the interviewee if they have anything to add. Any additional information is added to the interview notes as required.

Thank person and leave.
