# Supplementary material for: From research to real-life implementation: an evaluation of the scale up of a guided digital mental health intervention in Lebanon: Step-by-Step
Source: Front Public Health. 2025 Nov 11;13:1665093. doi: 10.3389/fpubh.2025.1665093 (PMC12643871; doi:10.3389/fpubh.2025.1665093)
Supplement: Supplementary file 5 [file Data_Sheet_5.DOCX]

# ANNEX 5

## Full list of session completion and drop-out rates.

| ***Drop-out at different stages of the project*** | | | |  |  |
| --- | --- | --- | --- | --- | --- |
|  | **N** | **Drop-out stage** | | **Total drop-out** | |
|  |  | **N** | **%** | **N** | **% of people completed baseline** |
| **Signed up for SbS** | **2429** |  |  |  |  |
| **Completed baseline** | **1942** | **487** | **20** |  |  |
| **Completed onboarding** | **1166** | **776** | **40** | **776** | **40** |
| **S1P1_STARTED** | **1084** | **82** | **7** | **858** | **44.2** |
| **S1P1_FINISHED** | **870** | **214** | **19** | **1072** | **55.2** |
| **S1P2_STARTED** | **776** | **94** | **11** | **1166** | **60.0** |
| **S1P2_FINISHED** | **742** | **34** | **4** | **1200** | **61.8** |
| **S1P3_STARTED** | **715** | **27** | **4** | **1227** | **63.2** |
| **S1P3_FINISHED** | **684** | **31** | **4** | **1258** | **64.8** |
| **S2P1_STARTED** | **560** | **124** | **18** | **1382** | **71.2** |
| **S2P1_FINISHED** | **543** | **17** | **3** | **1399** | **72.0** |
| **S2P2_STARTED** | **505** | **38** | **7** | **1437** | **74.0** |
| **S2P2_FINISHED** | **492** | **13** | **3** | **1450** | **74.7** |
| **S2P3_STARTED** | **471** | **21** | **4** | **1471** | **75.7** |
| **S2P3_FINISHED** | **446** | **25** | **5** | **1496** | **77.0** |
| **S3P1_STARTED** | **392** | **54** | **12** | **1550** | **79.8** |
| **S3P1_FINISHED** | **378** | **14** | **4** | **1564** | **80.5** |
| **S3P2_STARTED** | **370** | **8** | **2** | **1572** | **80.9** |
| **S3P2_FINISHED** | **357** | **13** | **4** | **1585** | **81.6** |
| **S3P3_STARTED** | **351** | **6** | **2** | **1591** | **81.9** |
| **S3P3_FINISHED** | **335** | **16** | **5** | **1607** | **82.7** |
| **S4P1_STARTED** | **305** | **30** | **9** | **1637** | **84.3** |
| **S4P1_FINISHED** | **298** | **7** | **2** | **1644** | **84.7** |
| **S4P2_STARTED** | **294** | **4** | **1** | **1648** | **84.9** |
| **S4P2_FINISHED** | **295** | **1** | **0** | **1647** | **84.8** |
| **S5P1_STARTED** | **271** | **24** | **8** | **1671** | **86.0** |
| **S5P1_FINISHED** | **263** | **8** | **3** | **1679** | **86.5** |
| **S5P2_STARTED** | **259** | **4** | **2** | **1683** | **86.7** |
| **S5P2_FINISHED** | **254** | **5** | **2** | **1688** | **86.9** |
| **S5P3_STARTED** | **236** | **18** | **7** | **1706** | **87.8** |
| **S5P3_FINISHED** | **230** | **6** | **3** | **1712** | **88.2** |

Note s= session, P=part. There were five SbS sessions, each with multiple parts.
